# A Novel Autosomal Recessive *GJA1* Missense Mutation Linked to Craniometaphyseal Dysplasia

**DOI:** 10.1371/journal.pone.0073576

**Published:** 2013-08-12

**Authors:** Ying Hu, I-Ping Chen, Salome de Almeida, Valdenize Tiziani, Cassio M. Raposo Do Amaral, Kalpana Gowrishankar, Maria Rita Passos-Bueno, Ernst J. Reichenberger

**Affiliations:** 1 Department of Reconstructive Sciences, Center for Regenerative Medicine and Developmental Biology, University of Connecticut Health Center, Farmington, Connecticut, United States of America; 2 Department of Oral Health and Diagnostic Sciences, University of Connecticut Health Center, Farmington, Connecticut, United States of America; 3 Medical Genetics Service, Centro Hospitalar de Lisboa, Central, Portugal; 4 Universidade Estadual Vale do Acaraú, Brazil; 5 Instituto de Cirurgia Plástica Cranio-facial - SOBRAPAR, Campinas, Brazil; 6 Department of Medical Genetics, Kanchi Kamakoti Childs Trust Hospital, Chennai, Tamil Nadu, India; 7 Instituto de Biociencias, Universidade de Sao Paulo, São Paulo, Brazil; Innsbruck Medical University, Austria

## Abstract

Craniometaphyseal dysplasia (CMD) is a rare sclerosing skeletal disorder with progressive hyperostosis of craniofacial bones. CMD can be inherited in an autosomal dominant (AD) trait or occur after de novo mutations in the pyrophosphate transporter *ANKH*. Although the autosomal recessive (AR) form of CMD had been mapped to 6q21-22 the mutation has been elusive. In this study, we performed whole-exome sequencing for one subject with AR CMD and identified a novel missense mutation (c.716G>A, p.Arg239Gln) in the C-terminus of the gap junction protein alpha-1 (*GJA1*) coding for connexin 43 (Cx43). We confirmed this mutation in 6 individuals from 3 additional families. The homozygous mutation cosegregated only with affected family members. Connexin 43 is a major component of gap junctions in osteoblasts, osteocytes, osteoclasts and chondrocytes. Gap junctions are responsible for the diffusion of low molecular weight molecules between cells. Mutations in Cx43 cause several dominant and recessive disorders involving developmental abnormalities of bone such as dominant and recessive oculodentodigital dysplasia (ODDD; MIM #164200, 257850) and isolated syndactyly type III (MIM #186100), the characteristic digital anomaly in ODDD. However, characteristic ocular and dental features of ODDD as well as syndactyly are absent in patients with the recessive Arg239Gln *Cx43* mutation. Bone remodeling mechanisms disrupted by this novel *Cx43* mutation remain to be elucidated.

## Introduction

Craniometaphyseal dysplasia (CMD; MIM #123000) is a rare genetic disorder affecting the skeleton with progressive hyperostosis of craniofacial bones and abnormal modeling of tubular bones. Craniofacial abnormalities include wide-set eyes, wide nasal bridge, paranasal bossing and prominent mandible. The main feature leading to morbidity is hyperostosis of cranial bones, which can lead to increased intracranial pressure and narrowing of neural foramina [1,2,3,4]. Nerve damage can lead to facial palsy, blindness and deafness. Increased bone formation can further lead to Chiari malformation, compression of the spinal cord and syringomyelia [5,6,7]. The metaphyses of long bones are widened and undertrabeculated, possibly due to insufficient bone remodeling by dysfunctional osteoblasts and osteoclasts [8,9].

Many CMD cases described so far are inherited as an autosomal dominant (AD) trait [10,11,12,13] or occur as *de novo* mutations [12,13,14,15,16]. Until now all CMD mutations have been found in the human progressive ankylosis gene (*ANKH*). *ANKH* encodes for a ten-span transmembrane protein associated with pyrophosphate transport [17]. Most of the *ANKH* mutations are located in cytoplasmic domains close to the C-terminus [12,13]. A CMD knock-in mouse model carrying a Phe377del mutation in *ANKH* develops many of the features that are characteristic for CMD patients and has been shown to exhibit impaired osteoblastogenesis and osteoclastogenesis [8,9].

Some pedigrees suggest an autosomal recessive (AR) mode of transmission [1,18,19,20,21] and a linkage study has identified a potential locus for the autosomal recessive form of CMD within a 7 cM interval on chromosome 6q21-22 [18]. However, a causative variant responsible for AR CMD could not be identified. Here we report the first gene mutation for autosomal recessive CMD in the gap junction gene *GJA1*, better known as CONNEXIN 43 (*CX43*), responsible for small molecule transport.

## Materials and Methods

### Ethics Statement

This study was approved by the Institutional Review Board at UCHC and the Ethics Committees at Kanchi Kamkoti, Childs Trust Hospital, University of Campinas, Ethics Commission for Health of the Hospital Center of Lisboa Central and Institute of Biosciences, University of Sao Paolo. Written informed consent had been obtained from study participants and parents of minors. Families and individuals included in this study were selected based on absence of mutations in ANKH and a potential recessive inheritance pattern. The data described here cannot be deposited to a public repository due to issues of patient confidentiality. Data can be shared with other investigators upon request and subject to clearance from our Institutional Review Board.

### Whole-Exome Sequencing

Genomic DNA (gDNA) was extracted from blood (Gentra Puregene; QIAGEN Inc., Valencia, CA) or saliva (OraGene saliva kit; DNA Genotek Inc., Kanata, Ontario, Canada) and submitted to Otogenetics Corporation (Norcross, GA USA) for exome capture and sequencing.

Briefly, gDNA was subjected to agarose gel electrophoresis and OD ratio tests to confirm the purity and concentration prior to fragmentation by a Covaris fragmenter (Covaris, Inc., Woburn, MA). Fragmented gDNAs were tested for size distribution and concentration using an Agilent Bioanalyzer 2100 (Agilent Technologies, Santa Clara, CA) and Nanodrop (Thermo, Fisher Scientific, Pittsburgh, PA). Sequencing libraries were prepared using NEBNext reagents (New England Biolabs, Ipswich, MA) and the resulting libraries were subjected to exome enrichment using the TruSeq Exome Enrichment Kit (Illumina, Inc., San Diego, CA) following manufacturer’s instructions. Enriched libraries were tested for enrichment by qPCR and for size distribution and concentration with Agilent Bioanalyzer 2100. The samples were then sequenced on an Illumina HiSeq2000, which generated paired-end reads of 100 nucleotides.

### Sequencing Alignment, Variant Calling and Annotation

Alignment and variant calling were evaluated on the DNAnexus platform (DNAnexus, Inc., Mountain View, CA). Burrows-Wheeler Aligner (BWA, version 0.5.9-r16) was used to align the sequence reads to the human reference genome GRCh37 (hg19). Subsequently, Nucleotide-Level Variation Analysis was used to identify differences in the sample genome with respect to the reference genome. Differences include single- and multi-nucleotide polymorphisms (SNPs, MNPs), insertions and/or deletions (indels). Resulting variant calls were first filtered for confidence calls originating from bidirectional sequence reads using a quality threshold of ≥30 and read depth ≥10. Variants were then prioritized for further filtering and retained for further analysis when a variant appeared as a homozygous and non-synonymous, i.e. altering an amino acid. Selected variants were then compared to public databases. All variants present in the dbSNP database (version 135), 1000 Genomes Project and NHLBI Exome Sequencing Project Exome Variant Server (EVS) were excluded.

### Mutation Validation Analysis

Nucleotide changes in the candidate gene *CX43* were numbered corresponding to their position in *CX43* mRNA (Ensembl gene ID: ENSG00000152661). Verification of the c.716G>A candidate variant was performed by Sanger sequencing of exon 2 of *CX43* using PCR primers GJA1-F (5’-TTCTGGGTCCTGCAGATCAT-3’) and GJA1-R (5’- TCTTGATGCTTTCAAGCCTGT-3’) resulting in a 944 bp product. All available family members were tested for cosegregation of the candidate variant with the disease phenotype. PCR products containing the variant of interest were purified with ExoSAP-IT (Affymetrix, Santa Clara, CA) and sequenced by Sanger sequencing using ABI PRISM 3730xl DNA Analyzers with standard protocol (Genewiz, South Plainfield, NJ). The functional effect of a missense variant was evaluated using in silico prediction tools PolyPhen2, SIFT, Mutation Taster and SNAP. Sequence alignment among different species was performed with ClustalW2.

## Results and Discussion

**Figure 1:**
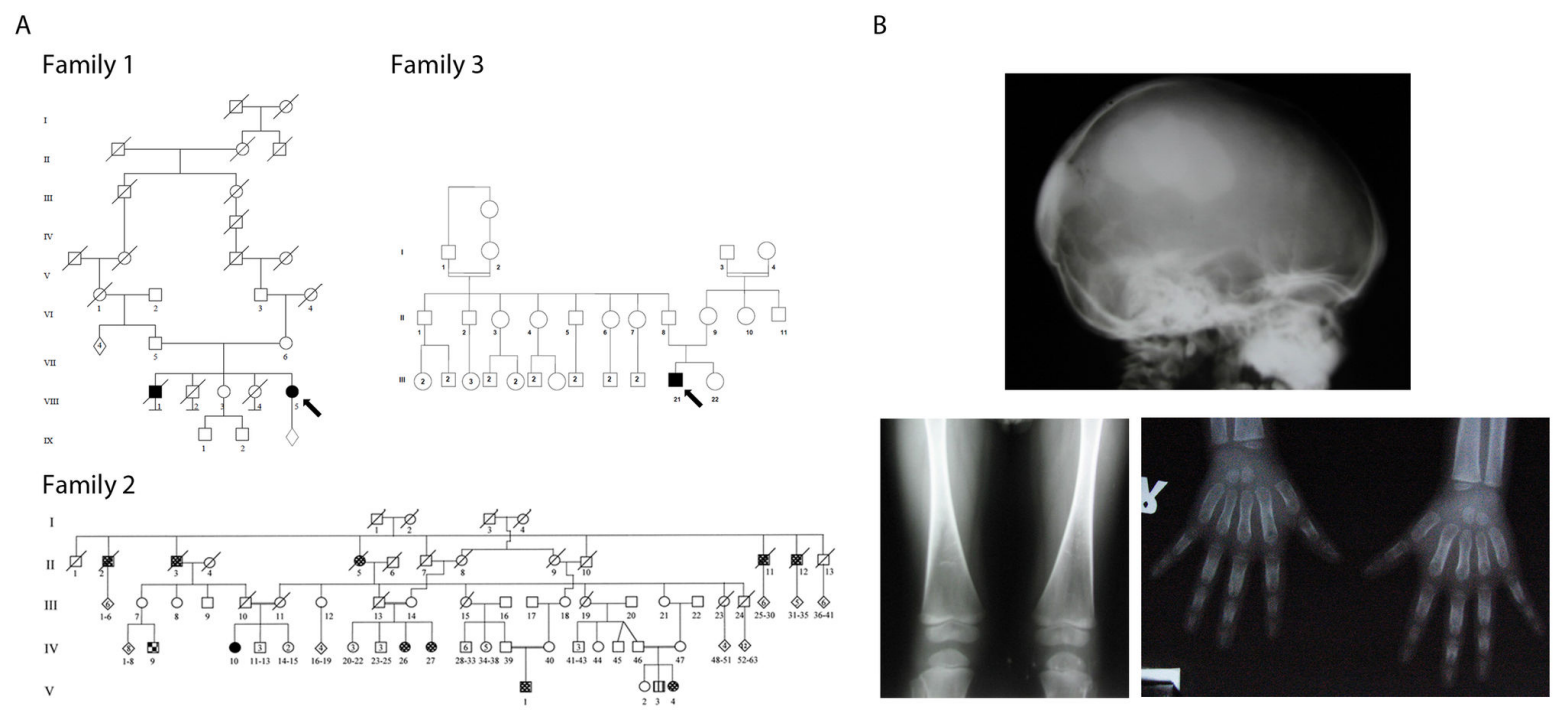
Pedigree information and case description. (A) Pedigrees of Families 1, 2 and 3 with mutation in the novel CMD gene *GJA1*. Proband DNA from Family 1 was used for exome sequencing (Arrow). (B) Radiographic images of proband for Family 3 show hyperostosis of cranial base and facial bones, femoral flaring and undertrabeculation of metaphyses with dense diaphyseal bone consistent with findings in Family 2. Diffuse widening of the proximal and medial phalanges are consistent with findings in CMD patients from Family 2 [18].

**Figure 2:**
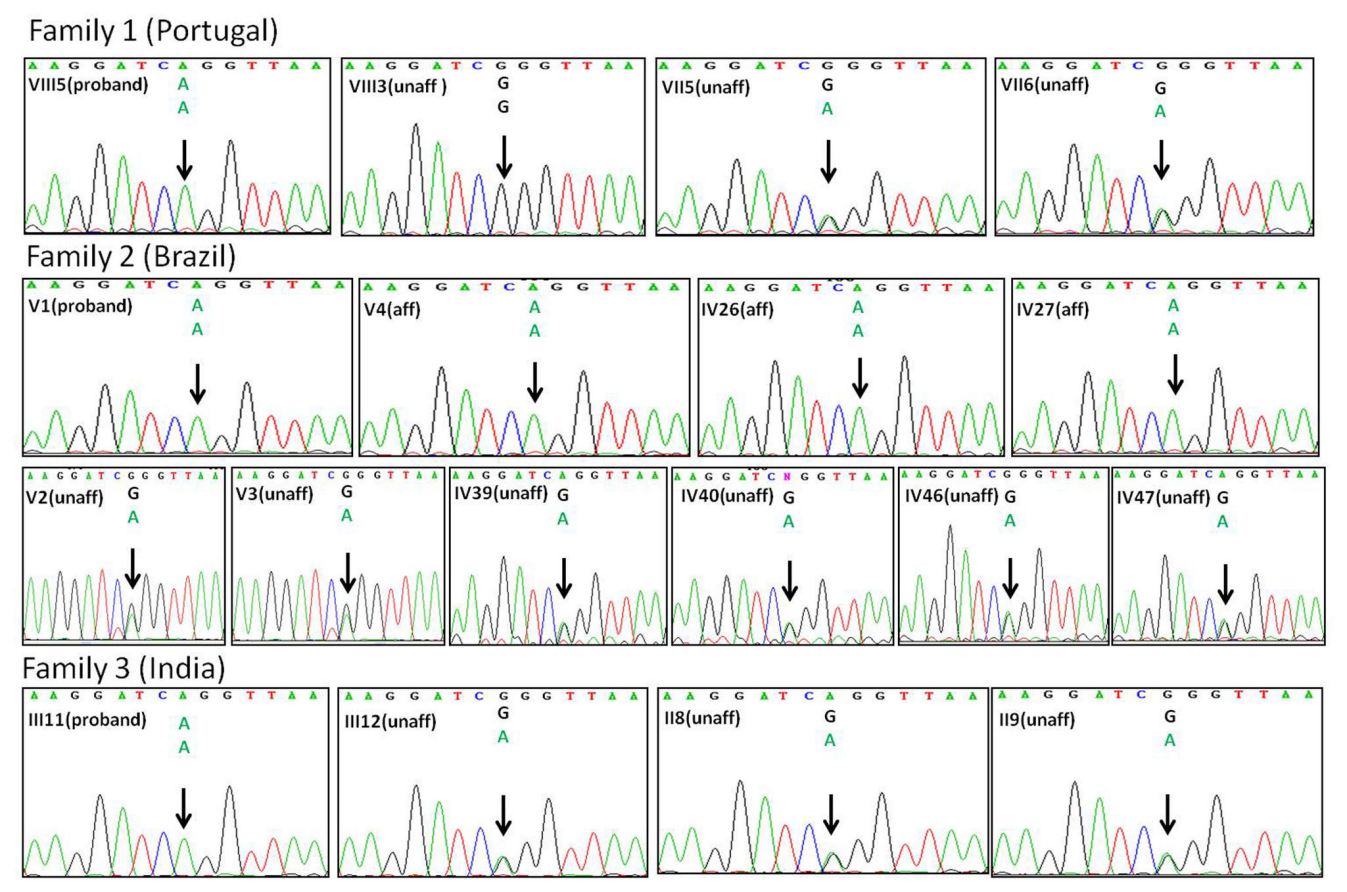
Confirmatory sequencing. Sanger sequencing to confirm exome sequencing data with a mutation in the CONNEXIN 43 gene *GJA1* in position c.716G>A (p.Arg239Gln). Data shown for Family 1 (VIII5 proband; VIII3 unaffected sibling; VII5 and VII6 heterozygous parents), Family 2 (V1 proband, V4 affected cousin; IV26 and IV27afffected aunts; V2 and V3 unaffected cousins; IV39 and IV40 heterozygous parents of V1; IV46 and IV47 heterozygous parents of V4) and Family 3 (III11 proband; III12 unaffected sibling; II8 and II9 heterozygous parents).

We performed exome sequencing on proband VIII5 from the consanguineous Family 1 (Figure 1) at an average depth of 78x using a TruSeq Exome 62Mb Enrichment kit and HiSeq2000 sequencer (Illumina). 20,794 genes were targeted which include 201,121 exons in total. A total of 163,463 genetic variants, including SNPs and indels were identified. Out of 46,866 homozygous variants 4,895 were non-synonymous. Only one of these variants, a G to A tansversion (c.716G>A) in *GJA1* (RefSeq NM_000165.3) (Figure 2), which was novel according to dbSNP, the Human Gene Mutation Database (HGMD), 1000 Genomes Project (approx. 1,000 individuals) and National Heart, Lung, and Blood Institute (NHLBI) Exome Sequencing Project (ESP; approx. 2,200 individuals) data colocalized with the putative recessive CMD locus on chromosome 6q21–q22. *GJA1* codes for the gap junction alpha 1 protein and is also known as *CONNEXIN 43*, *CX43* (MIM #121014).

**Figure 3:**
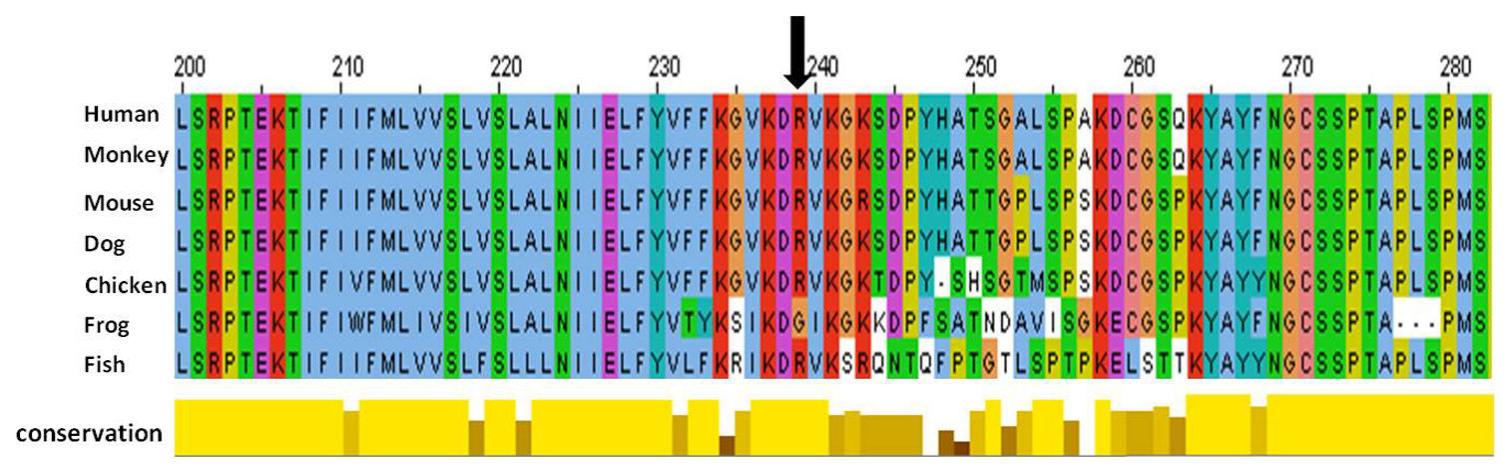
Phylogenetic comparison of *GJA1* across species. Position of the mutation within a highly conserved region indicated with arrow.

The non-synonymous missense variant in *GJA1* resulted in a homozygous amino acid substitution from Arginine to Glutamine (p.Arg239Gln; R239Q). This variant was predicted to be damaging by Mutation Taster and SNAP although SIFT and PolyPhen2 did not flag this mutation as damaging. The R239Q variant in CX43 is located in a phylogenetically highly conserved region, which has also been identified as a potential tubulin binding motif (Figure 3) [22]. This Arginine is conserved in 4 other connexins, CX37, (GJA4), CX40 (GJA5), CX30 (GJB6) and CX32 (GJB1), however, no mutations have been reported for Arginine at this position.

We then identified this variant in 4 affected individuals (V1 (proband), V4, IV26, IV27) of Brazilian Family 2 [18] and in the affected child of the Indian Family 3 (III11) by Sanger sequencing (Figure 2). The c.716G>A variant was absent in the unaffected sibling of family 1 (VIII3), who had a wild type genotype. The parents of all probands as well as two unaffected siblings in Family 2 and one unaffected sibling in Family 3 were heterozygous for the mutation. We found the same homozygous c.716G>A mutation in a fourth proband from Brazil diagnosed with CMD but with unknown family background (data not shown). The summary of all subject genotypes is shown in Table S1.

The affected individuals in Family 2 (V1, V4, V26 and IV27) were born to healthy consanguineous parents and showed relatively mild symptoms as described before [18] with nasal flattening and hypertelorism. 3D computed tomography and radiographs for V1 and V4 showed sclerosis of the cranial vault and cranial base. Facial bones were hyperostotic. All affected individuals in this study had difficulties breathing, probably due to hyperostosis, but none showed loss of vision or hearing loss. Affected members of this family displayed diaphyseal hyperostosis in limb bones with mild metaphyseal widening. Another AR disorder, a form of spondylocostal dysostosis (SD), also segregates in this family. Subject V3 with SD (indicated as striped square in Figure 1) showed normal skull and limb radiographs.

The proband of Family 3 (Figure 1B) is a 3-year-old male referred to the genetics clinic for epiphora (L) and dysmorphic facial features. His height was 96 cm (50th centile) and head circumference was 50 cm (50th–85th centile). He had relative macrocephaly, hypertelorism, an unusual thick bony wedge over the bridge of the nose and a depressed and flattened nasal bridge. The ophthalmological exam revealed secondary naso-lacrimal duct obstruction (bilateral). X-ray of skull revealed significant sclerosis of skull base and 3D MRI of paranasal sinuses and orbits showed bilateral dense sclerotic thickening in parietal, occipital and ethmoid bones as well as in the frontal process of maxillary and zygomatic bones. His serum calcium, phosphorus and serum alkaline phosphate levels were normal. The proband of Family 1 is unfortunately lost to follow up and detailed clinical information is no longer available.

**Figure 4:**
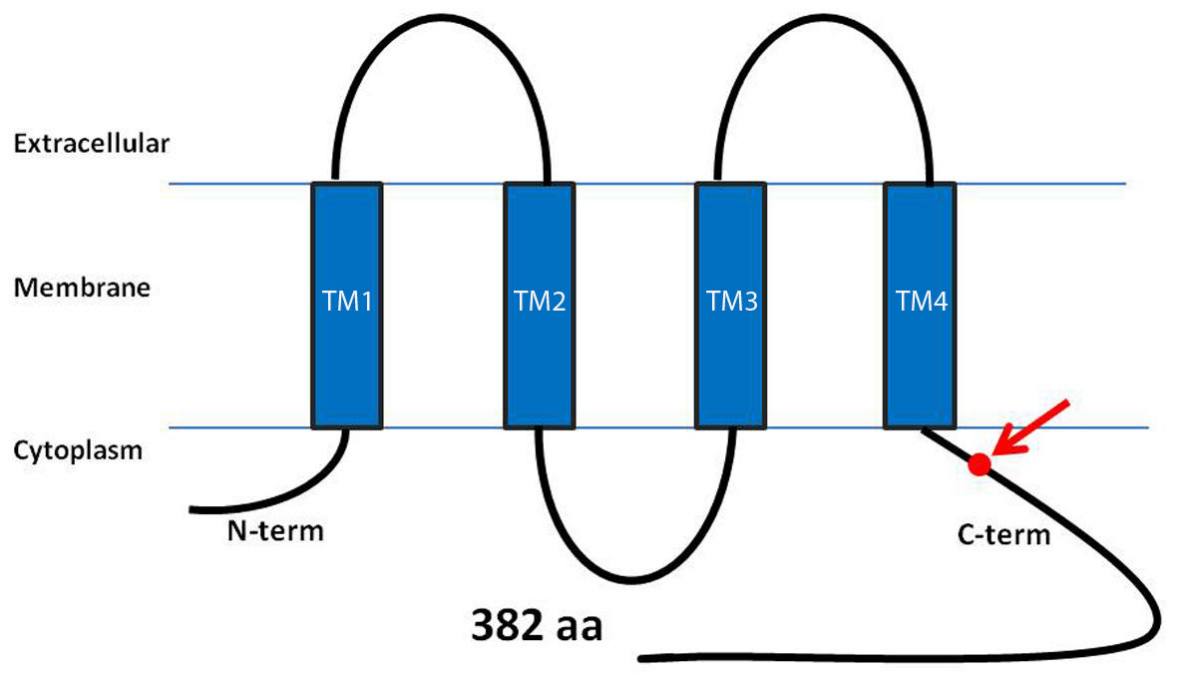
Domain structure of CONNEXIN 43. The CX43 protein consists of an intracellular amino acid domain (AT), four transmembrane domains (TM), two extracellular loops and one intracellular loop. The novel CMD mutation is indicated as a red dot in the carboxyl-terminal domain (CT).

CX43 is a major component of hemichannels and gap junctions. Two opposing hemichannels form a gap junction connecting the cytoplasm of neighboring cells to facilitate gap junctional intercellular communication (GJIC) via exchange of ions and molecules smaller than 1.2 kDa. The *GJA1* gene consists of 2 exons and encodes a transmembrane protein with four transmembrane domains (TM), two extracellular loops, one intracellular loop and intracellular amino- (N-term) and carboxyl-terminal ends (C-term) (Figure 4). The mutation identified in this study is located in exon 2 of the *CX43* gene and leads to a substitution in position 239 in the intracellular C-terminal domain proximal to the fourth transmembrane domain. At least three connexins, CX43, CX45 and CX46 are expressed in bone cells with CX43 being the most abundant. There is solid evidence for an important role of CX43 in skeletal development and the function and survival of osteoblasts and osteocytes [23,24,25,26]. Data from *in vitro* studies supported a role for CX43 and gap junctional intracellular communication during osteoblastic cell differentiation and coordinated cell responsiveness [27,28]. *In vivo* studies demonstrated that global ablation of *Gja1* in mice results in delayed skeletal ossification, craniofacial abnormalities and osteoblast dysfunction [29,30]. Tissue-specific *Gja1* ablation in osteoblasts results in accrual of a low peak bone mass and an attenuated response to the anabolic effects of parathyroid hormone (increase mineral apposition rate in response to PTH) despite increased osteoblast numbers, which suggests a functional defect in *CX43*-deficient bone-forming cells [31]. Osteoblast- and osteocyte-specific ablation of *CX43* using an osteocalcin-driven Cre-lox system resulted in bone loss due to increased bone resorption and osteoclastogenesis [32]. Mice lacking CX43 in osteocytes develop increased osteocyte apoptosis and empty lacunae in cortical bone [25].

Further proof of the critical role of CX43 in skeletal biology provides the identification of 65 mutations in *GJA1* as the cause of the predominantly autosomal dominant oculodentodigital dysplasia (ODDD; MIM # 164200). ODDD is characterized by craniofacial abnormalities, microphthalmia, tooth defects, and hand and foot abnormalities such as fifth finger camptodactyly, syndactyly of the fourth and fifth fingers, and missing phalanges of the toes. Other skeletal abnormalities include cranial hyperostosis, wide alveolar ridge of the mandible as well as broad tubular bones [33,34,35,36]. The fact that the CMD patients described in this study do not display the characteristic tooth defects, ocular involvement or camptodactyly / syndactyly of ODDD strongly suggests that their phenotype is consistent with CMD and not part of the ODDD spectrum.

Another rare genetic disorder, Hallermann–Streiff syndrome (HSS) is characterized by proportionate short stature, hypotrichosis, congenital cataracts, malformation of cranial and facial bones as well as dental anomalies (MIM #234100) and shares several clinical characteristics with ODDD. Recently, a single case with a homozygous *CX43* gene mutation p. R76H (c.227G>A) in a conserved region was found with overlapping phenotypes of HSS/ODDD [37]. Some digital anomalies such as type III syndactyly can be syndromic with ODDD although isolated type III syndactyly has been attributed to a *GJA1* mutation as well [38]. The only overlapping features of ODDD patients with CMD patients harboring a Arg239Gln mutation in *GJA1* are cranial hyperostosis and widening of tubular bones.

Most of the autosomal dominant CX43 mutations for ODDD that were studied in detail showed reduced channel function inhibiting wild type CX43 in a dominant negative manner [39,40]. It is therefore possible that the Arg239Gln mutation in *GJA1* also acts in a dominant negative manner by reducing the function of other connexin homo- or oligomeres or of interacting connection proteins. Recently, R76H and R33X mutations in CX43 have been reported as the cause for autosomal recessive ODDD by distinct mechanisms [41]. In vitro experiments showed that the R76H mutant was able to form functional gap junctions whereas the R33X mutant reduced the connexin gap junction plaques.

In addition to its role as intercellular channel CX43 has been shown to interact with intracellular structures and signaling molecules such as β-arrestins for PTH receptor/cAMP signaling in osteoblasts [42] and to affect cellular functions through its cytoplasmic C-terminal domain [43].

Gap junctions can serve as anchoring locations for microtubules thus regulating cellular activity associated with microtubule function. Microtubules are one of the major components of the cytoskeleton providing scaffolding, sequestration and delivery functions. They are involved in various signaling pathways such as Wnt, hedgehog (Hh), nuclear factor kB (NF-κB) and mitogen activated protein kinase K (MAPK) through diverse mechanisms [44,45,46,47,48,49]. These signaling pathways have also been implicated in skeletal development and skeletal homeostasis.

The CX43 C-terminal cytoplasmic domain harbors a highly conserved potential tubulin binding motif^234^ K G V KDR V K G K ^243^ [22] thus mediating the TGF-β signaling pathway via Smad2/3, which is a major player in osteoblast differentiation and osteoblast-induced osteoclastogenesis [50]. CX43 competes with Smad2/3 for binding to microtubules inducing release of Smad2/3 from microtubules [51] and can increase phospho-Smad2/3 accumulation in the nucleus leading to transcriptional activation of target genes.

The R239Q mutation in our CMD patients is located within this tubulin binding motif and we hypothesize that the amino acid change may affect binding to microtubules. In this case Smads may compete with microtubule binding and with their translocation to the nucleus thus altering the expression of osteoblastic genes regulated by Smads.

Therefore, it is possible that the *CX43* mutation causes CMD by either changing the tubulin binding site or by affecting signal transduction events via alteration of transport properties of gap junctions/hemichannels.

## Conclusions

In summary, our exome sequencing study identified a novel disease gene for the autosomal recessive form of craniometaphyseal dysplasia. The causative variant in the CONNEXIN 43 gene *GJA1* results in a disease-specific CMD phenotype demonstrating that CMD is a genetically heterogeneous disorder. It will be interesting to study any discernible clinical differences of the recessive CX43 form of CMD to the autosomal dominant form caused by ANKH mutations once more patients with *CX43* mutations have been identified for this very rare craniotubular disorder. It is also remains to be investigated whether any mechanistic interactions between CX43 and ANKH exist.

### URLs

1000 Genomes Browser: http://browser.1000genomes.org/

NHLBI Exome Variant Server: http://evs.gs.washington.edu/EVS/

The Human Gene Mutation Database (HGMD): http://www.hgmd.cf.ac.uk/ac/index.php

DbSNP: http://www.ncbi.nlm.nih.gov/projects/SNP/ ;

Online Mendelian Inheritance in Man (OMIM): http://www.ncbi.nlm.nih.gov/omim ;

SIFT: http://sift.jcvi.org/ ;

Polyphen2: http://genetics.bwh.harvard.edu/pph2/ ;

SNAP: http://www.rostlab.org/services/snap/ ;

MutationTaster: http://www.mutationtaster.org/ ;

ClustalW2: http://www.ebi.ac.uk/Tools/msa/clustalw2/.

### Accession numbers

The following accession codes were used to number nucleotide changes in *CONNEXIN 43*: Ensembl gene ID: ENSG00000152661.

We thank the families for participating in this study and for institutional support from the University of Connecticut Health Center and for NIH support # M01RR006192 to the UCHC CRC.

## Supporting Information

Table S1 Summary of AR CMD subjects in this study. (DOCX)
