# Supplementary material for: A Novel Autosomal Recessive GJA1 Missense Mutation Linked to Craniometaphyseal Dysplasia
Source: PLoS One. 2013 Aug 12;8(8):e73576. doi: 10.1371/journal.pone.0073576 (PMC3741164; doi:10.1371/journal.pone.0073576)
Supplement: Table S1 — (DOCX) [file pone.0073576.s001.docx]

**Supplementary Table S1:** Summary of AR CMD subjects in this study

| **Individual** | **Family No.** | **Original** | **Phenotype** | **Relatedness** | **R239Q genotype** |
| --- | --- | --- | --- | --- | --- |
| VIII5 | 1 | Portugal | Affected | Proband | HOMO |
| VIII3 | 1 | Portugal | Unaffected | Sibling | WT |
| VII5 | 1 | Portugal | Unaffected | Father | HET |
| VII6 | 1 | Portugal | Unaffected | Mother | HET |
| IV26 | 2 | Brazil | Affected | Aunt | HOMO |
| IV27 | 2 | Brazil | Affected | Aunt | HOMO |
| IV39 | 2 | Brazil | Unaffected | Father | HET |
| IV40 | 2 | Brazil | Unaffected | Mother | HET |
| IV46 | 2 | Brazil | Unaffected | Uncle | HET |
| IV47 | 2 | Brazil | Unaffected | Aunt | HET |
| V1 | 2 | Brazil | Affected | Proband | HOMO |
| V2 | 2 | Brazil | Unaffected | Cousin | HET |
| V3 | 2 | Brazil | Unaffected | Cousin | HET |
| V4 | 2 | Brazil | Affected | Cousin | HOMO |
| II8 | 3 | India | Unaffected | Father | HET |
| II9 | 3 | India | Unaffected | Mother | HET |
| III11 | 3 | India | Affected | Proband | HOMO |
| III12 | 3 | India | Unaffected | Sibling | HET |
